# Otoacoustic emissions from ears with spontaneous activity behave differently to those without: Stronger responses to tone bursts as well as to clicks

**DOI:** 10.1371/journal.pone.0192930

**Published:** 2018-02-16

**Authors:** W. Wiktor Jedrzejczak, Krzysztof Kochanek, Henryk Skarzynski

**Affiliations:** 1 Institute of Physiology and Pathology of Hearing, ul. M. Mochnackiego 10, Warsaw, Poland; 2 World Hearing Center, ul. Mokra 17, Kajetany, Nadarzyn, Poland; University of Leeds, UNITED KINGDOM

## Abstract

It has been reported that both click-evoked otoacoustic emissions (CEOAEs) and distortion product otoacoustic emissions (DPOAEs) have higher amplitudes in ears that possess spontaneous otoacoustic emissions (SOAEs). The general aim of the present study was to investigate whether the presence of spontaneous activity in the cochlea affected tone-burst evoked otoacoustic emissions (TBOAEs). As a benchmark, the study also measured growth functions of CEOAEs. Spontaneous activity in the cochlea was measured by the level of synchronized spontaneous otoacoustic emissions (SSOAEs), an emission evoked by a click but closely related to spontaneous otoacoustic emissions (SOAEs, which are detectable without any stimulus). Measurements were made on a group of 15 adults whose ears were categorized as either having recordable SSOAEs or no SSOAEs. In each ear, CEOAEs and TBOAEs were registered at frequencies of 0.5, 1, 2, and 4 kHz, and input/output functions were measured at 40, 50, 60, 70, and 80 dB SPL. Global and half-octave-band values of response level and latency were estimated. Our main finding was that in ears with spontaneous activity, TBOAEs had higher levels than in ears without. The difference was more apparent for global values, but were also seen with half-octave-band analysis. Input/output functions had similar growth rates for ears with and without SSOAEs. There were no significant differences in latencies between TBOAEs from ears with and without SSOAEs, although latencies tended to be longer for lower stimulus levels and lower stimulus frequencies. When TBOAE levels were compared to CEOAE levels, the latter showed greater differences between recordings from ears with and without SSOAEs. Although TBOAEs reflect activity from a more restricted cochlear region than CEOAEs, at all stimulus frequencies their behavior still depends on whether SSOAEs are present or not.

## Introduction

The first publication describing otoacoustic emissions (OAEs) dealt with cochlear responses evoked by clicks (i.e. click-evoked OAEs or CEOAEs) [1], followed shortly by descriptions of distortion product OAEs (DPOAEs) and spontaneous OAEs (SOAEs) [2]. In the same year, tone-burst-evoked OAEs (TBOAEs) were also described [3]. Both CEOAEs and TBOAEs are measured in a time window following a short stimulus, and are therefore classified as transient evoked OAEs (TEOAEs). OAEs can also be evoked by continuous tones and combination of tones, as in stimulus frequency OAEs (SFOAEs) and DPOAEs respectively.

Of all the types of evoked OAEs, the least researched are TBOAEs. They can be measured in all age groups: newborns [4], children [5], and adults [6,7]. TBOAEs can provide important information about basic cochlear function. They have been used to compare latencies of OAEs and auditory brainstem responses (ABRs) in order to test the theory of backward traveling waves [8,9]. Other investigators have used them to estimate loudness [10] and to measure local suppression effects in the cochlea [11–13]. To complete the picture, they can also be useful in the diagnosis of general hearing loss [14], in testing high frequency hearing deficits [15], and to improve hearing screening tests in newborns [16].

As with other types of evoked OAEs, TBOAEs are generally present in all subjects with normal hearing. Some fraction of normal individuals also stand out by having SOAEs. The prevalence of SOAEs depends on age as well as the measuring equipment and testing environment. For adults, prevalence has been reported as 40–70% [17–19], a figure which depends on the prevailing noise floor. Generally, the better the measurement conditions the higher the SOAE prevalence. Because SOAEs are of generally low level, a long averaging process is usually needed to measure them. However, it has been found that if SOAEs are synchronized to a stimulus, a higher level signal can be achieved. This feature can be used to develop a method of measurement called synchronized SOAEs (SSOAEs). It is typically based on using a click stimulus but with a longer acquisition window. Such a facility is usually available in commercial OAE equipment. Despite their different modes of acquisition, SOAEs and SSOAEs generally have similar properties, including frequency [20,21]. However, their prevalence is usually greater, i.e. up to 85% [22,23]. This increase in abundance is mostly related to the fact that the synchronizing stimulus excites two types of SSOAEs: stable sinusoidal ones, like ‘pure’ SOAEs, and exponentially decaying ones [24]. The decaying components are in fact difficult to distinguish from pure SOAEs as their decay might be longer than the recording window [24]. However, for practical purposes and in order to simplify the terminology, throughout this manuscript the term ‘ears with SSOAEs’ or ‘with spontaneous activity’ means ears with detectable SSOAEs.

Quite soon after the discovery of OAEs it was found that SOAEs or SSOAEs can have an effect on evoked OAEs [2]. In fact, it has subsequently been reported that all types of evoked OAEs are in some way affected by the presence of SOAEs, usually by showing higher amplitudes. This is the case for CEOAEs [21,25–27]; for DPOAEs [25,28]; for SFOAEs [29]; and for TBOAEs as well [30,31,32,33]. The dependence of OAE level on stimulus level–i.e. the input/output (I/O) functions of ears with and without SOAEs and SSOAEs–has been quite extensively investigated for CEOAEs [21,34] and DPOAEs [28,35]. However, to our knowledge there is only one report on such data for TBOAEs, and this only for a single frequency (1.5 kHz) [30].

An important OAE parameter is latency (i.e. the time from stimulus onset to the maximum of the waveform). The latencies of both CEOAEs and TBOAEs depend largely on their frequencies and stimulus levels (e.g. [9,36]), and with some hearing deficits the latencies may become longer (e.g. [37,38]). It has also been found that shifts in latency may occur when SSOAEs are present, an effect attributed to SSOAEs adding to the evoked OAE waveform, which causes a shift in the envelope maximum and hence the latency [39]. Initial findings were that the latency of TBOAEs shifts towards longer values with decreasing stimulus level [40,41]. In more recent work it was found that TBOAEs contain short latency (SL) and long latency (LL) components [42,43]. It appears that the latency does not actually shift with stimulus level but rather switches from the SL to the LL component. However, it has not been ascertained whether SL or LL latencies are affected by the presence of SSOAEs. The fact that OAE latency might be influenced by hearing status, and that SSOAEs are likely to depend on both SL and LL components, warrants an investigation of whether SSOAEs affect the latency, as well as the level, of TBOAEs.

The rationale for the present study rests on noting that, although TBOAEs are important (e.g. [9,15]), we know little about them. In particular, I/O data have been presented in only few studies with different sets of equipment, often custom-built [9,10,13,30,40,41]. The present study used a commercially available system, frequently used in research, in order to provide a standard baseline. Additional motivation came from previous TBOAE studies, especially of low-frequency (0.5 kHz) TBOAEs [33], where TBOAEs were affected by SSOAEs. A limitation of this previous study, however, was that the TBOAEs were evoked only by a single 80 dB SPL stimulus. Here the range is extended; moreover, it seemed important to investigate how the properties of evoked OAEs differ between ears with and without detectable SSOAEs. In this regard, we note that spontaneous activity is present in a substantial proportion of normal subjects and yet some authors have excluded ears with detectable SOAEs when analyzing TBOAEs (e.g. [41,44]).

The purpose of the present study was therefore to characterize the I/O functions of TBOAEs and to gauge how strongly their thresholds and levels are affected by the presence of SSOAEs. Considerations of possible multiple components meant that TBOAE latency was also a factor of interest. Finally, as a point of reference, CEOAEs were also measured, as their I/O characteristics and their dependence on spontaneous activity has been fairly well documented. Finally, the present work attempts to provide a benchmark for TBOAEs measured with a commercially available system, since the data in the literature (e.g. [15]) relate to older versions which use different stimulus settings.

## Material and methods

OAEs were recorded from both ears of a group of 15 adults (11 females, 4 males, age 25–35). All subjects underwent visual inspection of the ear canal and tympanic membrane of both ears followed by tympanometry, pure tone audiometry, and OAE measurements. All subjects had normal middle ear function (as assessed by 226 Hz tympanometry) and hearing thresholds equal to or better than 20 dB HL for all test frequencies from 0.25 to 8 kHz. The subjects gave written informed consent prior to participation. Research procedures were approved by the Ethics Committee of the Institute of Physiology and Pathology of Hearing, Poland.

### A. TBOAEs and CEOAEs

In each ear, a set of measurements were made: CEOAEs and TBOAEs at frequencies of 0.5, 1, 2, and 4 kHz. For each stimulus type, input/output (I/O) functions were measured for levels of 40, 50, 60, 70, and 80 dB peak equivalent (pe) SPL (the 40 dB level was the lowest level of stimulation available in the system).

OAEs were measured in low-noise ambient conditions (in a sound attenuating booth) using an ILO 292 system (Otodynamics Ltd) running software version 5.6. All protocols and stimuli parameters were those preset in the system. The only settings that could be changed were stimulus level and number of averages. The nonlinear averaging protocol was used (three stimuli of the same level and polarity, the fourth stimulus with three times higher magnitude and reversed polarity). The tone bursts were 2 ms long with equal rise/fall times and no plateau. Accordingly, they had the following number of cycles: 1 for 0.5 kHz, 2 for 1 kHz, 4 for 2 kHz, and 8 for 4 kHz. All recordings were made with an interstimulus interval of 20 ms. The initial part of the response was windowed automatically by the system to minimize stimulus artifacts. A cosine window was used with rise and fall times of 2 ms. For click stimuli, the window was 2.5–20 ms, for tone bursts of 0.5 and 1 kHz it was 4–20 ms, and 3–20 ms for 2 and 4 kHz tone bursts. The responses were averaged into two buffers, A and B. The estimate of the signal was (A+B)/2 and that of the noise (A–B)/2. The averaging process was ended if the global SNR was higher than 6 dB and at least 260 averages had been made (260 is the default setting for the ILO). If an SNR of 6 dB had not been achieved, averaging was continued until 6 dB or 780 averages was reached. Because the tone bursts were of short duration, spectral splatter meant that a broad range of frequencies was stimulated (typically ±0.9 kHz around the center frequency).

OAE response levels were calculated in terms of global values (the complete unfiltered signal) and half-octave-band values (centered on 0.5, 1, 2, and 4 kHz). Only responses that had an SNR of at least 3 dB were analyzed.

It should be noted that half-octave band levels were typically higher for TBOAEs than for CEOAEs, an effect mainly related to stimulus calibration [41]. In more detail, the calibration differences arose because both tone bursts and clicks are calibrated in dB pe SPL. Therefore, if one stimulus (e.g. a tone burst in the case of a TBOAE measurement) is spread over a narrower frequency band than the other (e.g. a click for CEOAEs), and both have the same dB pe SPL level, then the spectrum level of the narrower frequency band stimulus will be higher. In practice, it was difficult to investigate this effect directly since the stimuli are not recorded by the ILO 292 system and are therefore unavailable for analysis. However, this issue has been addressed more fully in an earlier study [41].

### B. SSOAEs

Additionally, in each ear recordings of synchronized spontaneous OAEs (SSOAEs) were made. SSOAEs were acquired using the inbuilt technique provided by the ILO 292 equipment. The stimulus was a click (70 dB pe SPL) but the interstimulus interval in this case was 80 ms, and the response was analyzed in a window of 21–80 ms, i.e. after the evoked part of the response had faded away. An ear was classified as “with SSOAEs” when at least one long-lasting peak exceeding the noise floor by 3 dB was found in the SSOAE spectrum [18,45].

**Fig 1:**
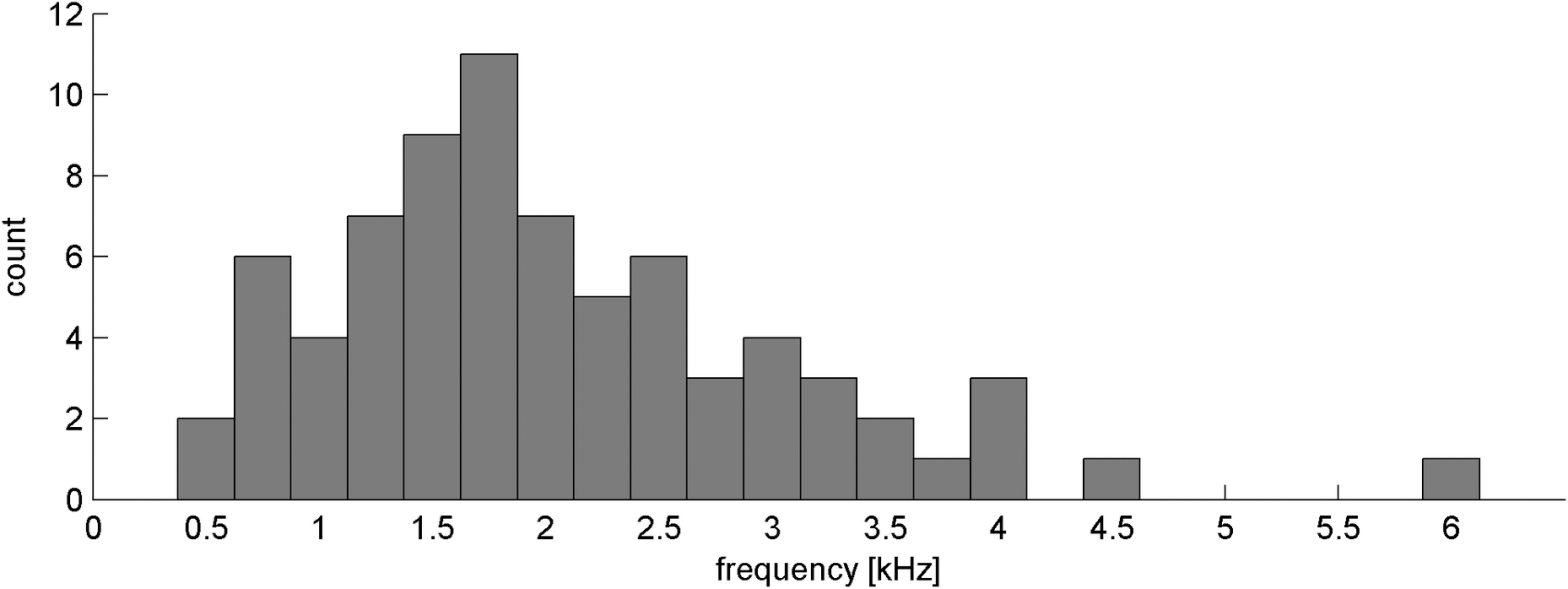
Histogram of SSOAEs frequencies.

The frequency distribution of SSOAEs is shown in Fig 1. The dataset was divided into two subsets: ears with (16 ears) and without SSOAEs (14 ears). In terms of audiometric thresholds, at no frequency was there any statistically significant difference between ears with and without SSOAEs.

### C. Latency estimates

**Fig 2:**
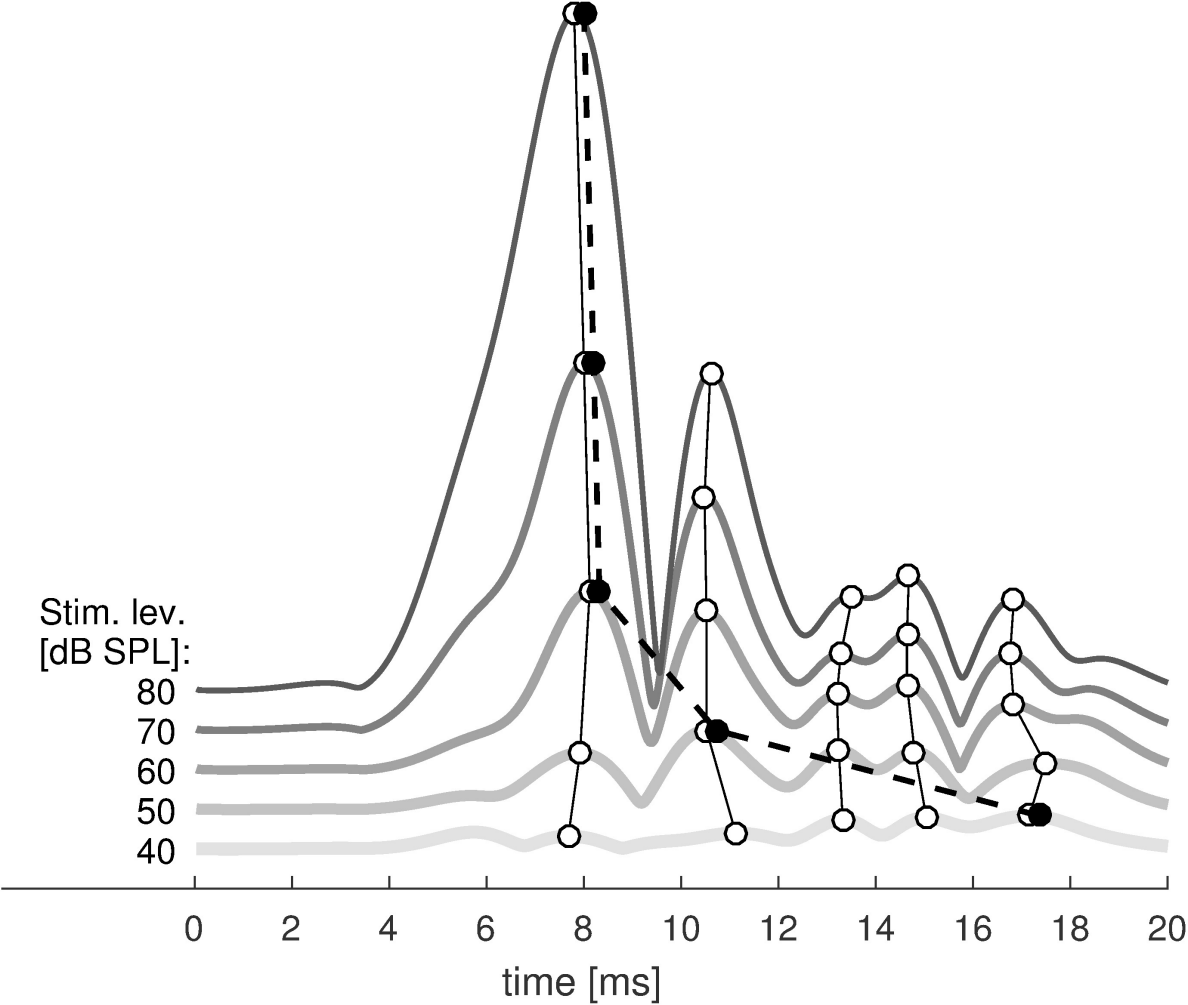
Example of the latency determination procedure. Envelopes of a 1 kHz TBOAE from a single subject are shown on a linear scale for stimulus levels from 40 to 80 dB. The white circles connected with solid lines mark the same maxima across different stimulus levels. Black circles (slightly offset for clearer presentation) connected with a dashed line mark the highest response for each stimulus level (the WS latency).

OAE latency is often estimated using various time–frequency analysis methods (e.g. [46–49]). However in some recent studies of TBOAEs, a much simpler approach based on the Hilbert transform has been used (e.g. [42,43]). Here, a similar approach was used. The signals were filtered in a half-octave band around the center frequency of the particular tone burst (i.e. 0.5, 1, 2, 4 kHz). Only signals that surpassed the noise by 3 dB were further analyzed. In the next step, the envelope of the signal was calculated using the Hilbert transform. Finally, maxima in the envelope were taken to be the latencies of the different components. Of interest was the latency of the maximum amplitude of the whole signal (WS), and latencies of the SL and LL components. An example of latency estimation is shown in Fig 2. Because the current study focused on the I/O characteristics of TBOAEs, a problem arose that the latency for the envelope maximum may change for different levels, i.e. switch between SL and LL components (e.g. [43]). Although we found that the latency of the maximum of the signal (WS latency) changed with stimulus level, we found that the latency of the individual components remained quite stable (see Fig 2). Here, the latencies of the individual components do not appear to shift, but the latency of the maximum switches to later components as stimulation levels become lower.

### D. Statistical analysis

The statistical significance of the mean difference between groups was evaluated for all parameters using a *t*-test or, when datasets did not have a normal distribution, a Wilcoxon rank sum test. For some analyses, Pearson correlations and simple linear regression were calculated. The goodness of fit of the linear regression was estimated by means of *R*^2^ (the ratio between the sum of the squares of the regression and the total sum of the squares). In all analyses, a 95% confidence level (*p* < 0.05) was chosen as the criterion of significance. When conducting multiple comparisons, *p* values were adjusted using the Benjamini–Hochberg procedure to control for false discovery rates [50].

## Results

### A. TBOAE levels

The TBOAEs were analyzed both in terms of global values (the unfiltered signal) and for the four half-octave bands centered around frequencies of 0.5, 1, 2, and 4 kHz.

**Fig 3:**
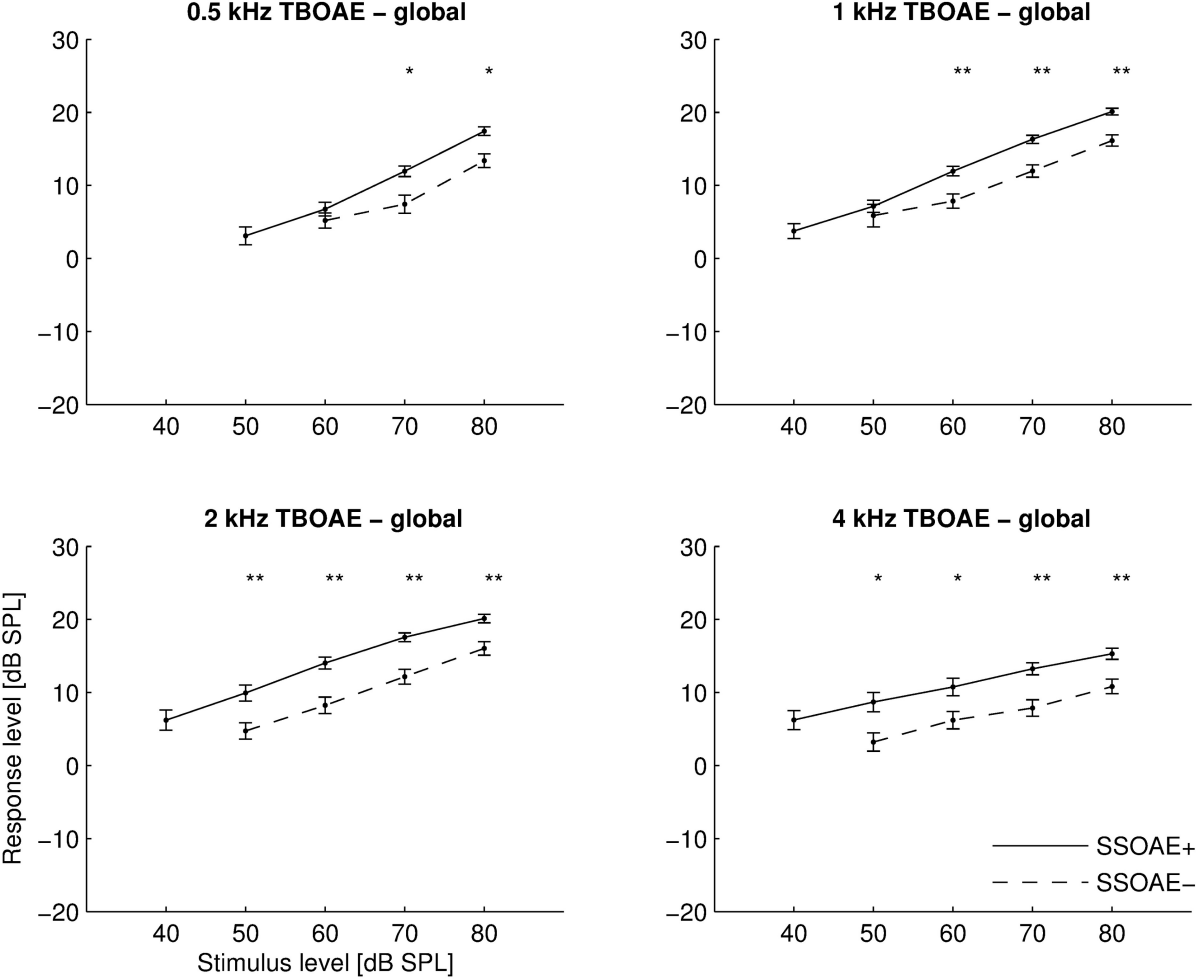
Mean input/output (I/O) functions measured as global response levels of TBOAEs for 4 different stimulus frequencies (center frequency given above each plot). Results are shown for ears with SSOAEs (solid line) and ears without SSOAEs (dashed line). Error bars indicate standard errors; asterisks at top of each panel indicate statistically significant differences (*–*p*<0.05; **–*p*<0.01).

Looking first at global (broadband) values, Fig 3 shows mean global response levels for TBOAEs from all tested ears, divided into groups with and without SSOAEs (designated as SSOAE+ and SSOAE–respectively). Each panel represents responses to a tone burst of different frequency. Only responses with SNR exceeding 3 dB were taken into account. The average values were calculated only when at least half of the ears in a given group fulfilled this condition (and so for some low-level stimuli mean values are not shown). TBOAEs had higher levels in ears with SSOAEs than in ears without SSOAEs, and the differences were significant (*p*<0.05) for most stimulus levels. The difference was around 3–5 dB for most TBOAE frequencies and stimulus levels. TBOAE global levels grew with stimulus level forming nearly straight line on a log-log scale, although for ears with SSOAEs a slight saturation is visible in Fig 3 for stimulation at 70 and 80 dB SPL and 2 kHz. Generally, for ears with SSOAEs, the response level grew by an average of 4.8 dB when the stimulus increased from 40 to 50 dB, and by 3.5 dB when it increased from 70 to 80 dB. For ears without SSOAEs, the behaviour was similar, except a 70 to 80 dB stimulus change produced a response 4.3 dB larger on average. For 0.5 kHz stimulation, the I/O slope was greater than at other frequencies; the signal rose above the noise at 50 dB for ears with SSOAEs and at 60 dB for ears without SSOAEs. For the other TBOAEs (1–4 kHz), the signal rose above the noise at 40 dB for ears with SSOAEs and at 50 dB for ears without SSOAEs.

**Fig 4:**
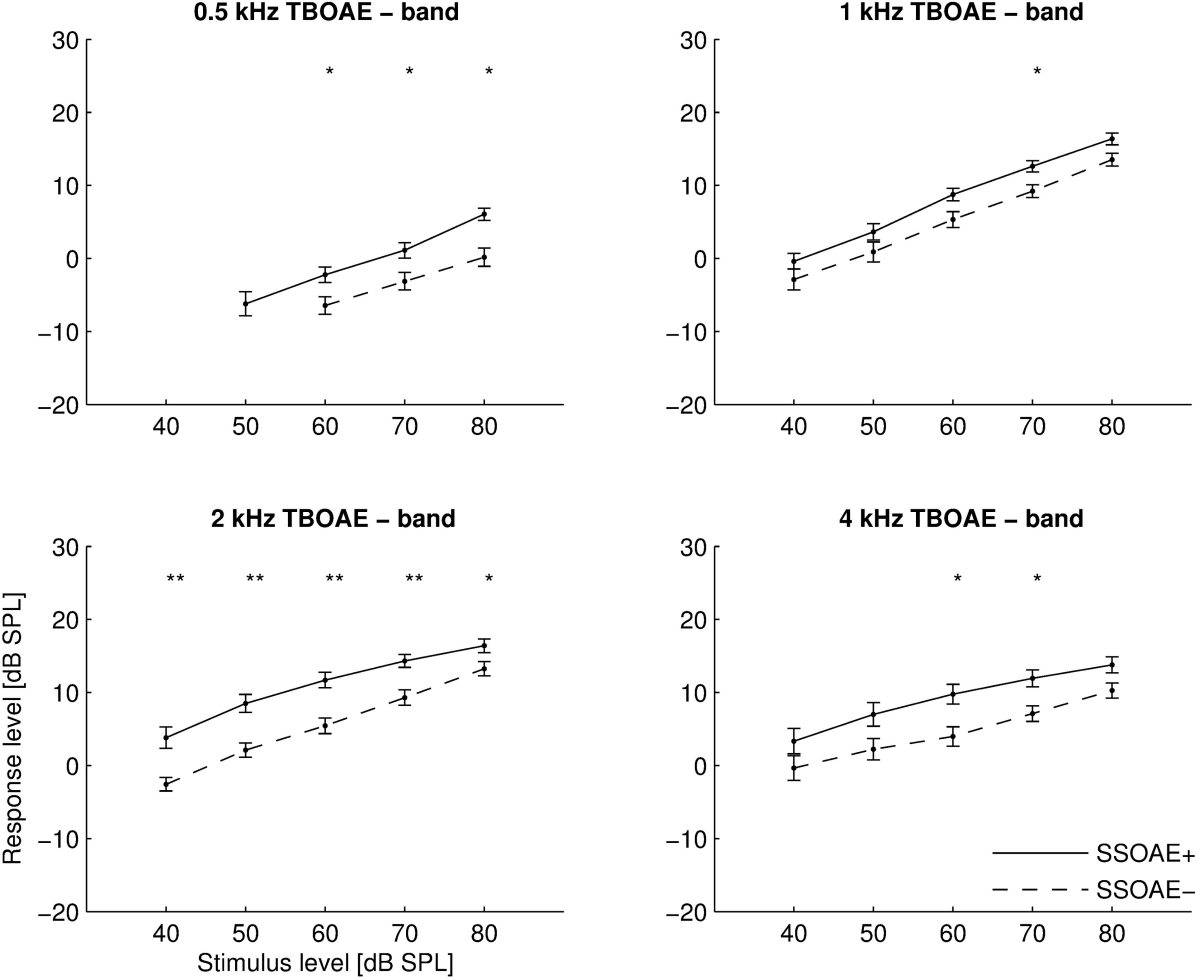
Mean I/O functions of TBOAEs measured in 4 half-octave bands (centre frequencies at top). Other details as in Fig 3.

Fig 4 presents the mean I/O functions of the same TBOAE datasets but this time analyzed into half-octave bands around the center frequency of each tone burst. These results more directly reflect the intrinsic properties of TBOAEs, since global values include components of the signal (e.g. SSOAEs) derived from frequencies some distance away from the frequency of stimulation. In addition, when analyzing in half-octave bands, it is possible to detect more responses above the noise at low stimulus levels, so the differences between ears with and without SSOAEs become less pronounced. Nevertheless, the differences are still visible and significant (*p*<0.05) for several stimulus levels. The difference reaches a maximum of about 6 dB for the 2 kHz TBOAE at stimulus levels of 40–50 dB. The differences are also visible at other frequencies of stimulation, although they are generally smallest for 1 kHz. For ears with SSOAEs, the response level grew by an average 4.8 dB when the stimulus increased from 40 to 50 dB, and by 3.1 dB when it increased from 70 to 80 dB. For ears without SSOAEs, it was about 0.5 dB higher for all increases in stimulus level (except that a change in stimulus level from 70 to 80 dB gave a response 4.1 dB larger on average).

### B. TBOAEs vs CEOAEs

**Fig 5:**
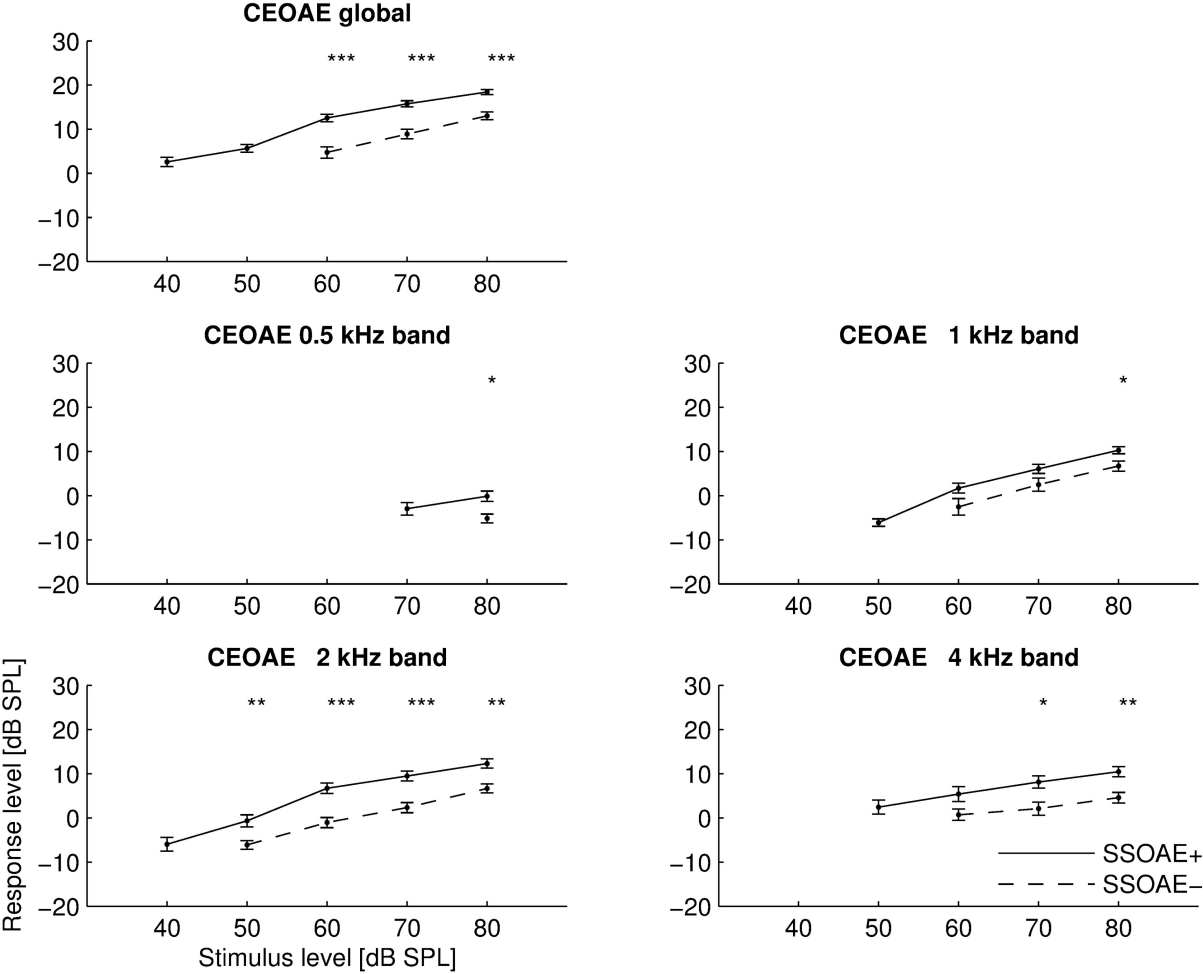
Mean I/O functions for CEOAEs measured globally (top plot) and in half-octave bands (four bottom plots). Asterisks at top of each panel indicate statistically significant differences (*–p<0.05; **–*p*<0.01; ***–*p*<0.001). Other details as in Fig 3.

CEOAE I/O functions were also calculated for comparison with I/O functions for TBOAEs, and the results are shown in Fig 5 for both global and half-octave frequency bands. Compared to the TBOAE I/O functions shown in Figs 3 and 4, the I/O functions for CEOAEs are very similar, but lower by about 5 dB. There is also slightly more saturation starting at the 60 dB stimulus level, which is most evident for global values and for the 1 and 2 kHz half-octave bands for SSOAE+ ears. The I/O function for the 0.5 kHz band shows very small responses. For SSOAE–ears the responses at this frequency were measured only for 80 dB SPL stimuli. Compared to TBOAEs, the effect of SSOAEs on CEOAEs is greater. The difference between the two curves in Fig 5 is about 5 dB for most frequencies and levels, and sometimes reached as much as 8 dB.

**Fig 6:**
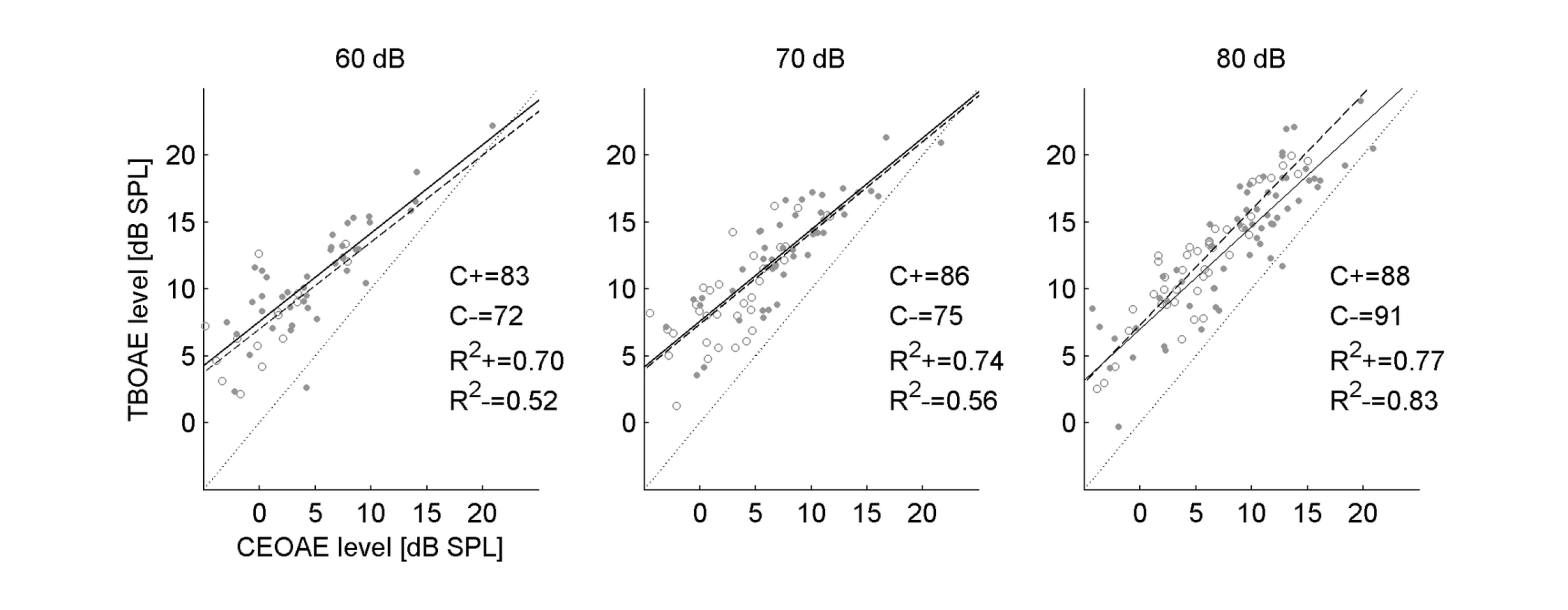
TBOAE level plotted against CEOAE level, both measured in half-octave bands, for 60, 70, and 80 dB stimuli. The data are plotted for ears with SSOAEs (dots) and without SSOAEs (circles). Linear fits for each group are also shown (solid: with SSOAEs; dashed: without SSOAE). Dotted line shows y = x line as reference. Percent correlations and R2 values for fits shown (‘+’, with SSOAEs; ‘–′, without SSOAEs).

To look closer at the differences between TBOAEs and CEOAEs, correlation and simple linear regression analysis was performed, and the results are shown in Fig 6. The dataset was again divided into ears with and without SSOAEs, and analyses performed in half-octave bands. The data for all stimulus frequencies was pooled and only responses exceeding the noise by 3 dB were included. This meant there were few data points for the 0.5 kHz band, as CEOAEs at this frequency rarely exceed the noise floor. Similarly, there were few or no data points for stimulus levels of 40 and 50 dB; consequently Fig 6 only shows data for stimuli of 60, 70, and 80 dB. Even though CEOAEs saturated at lower levels than TBOAEs, Fig 6 shows that a linear model fits the data well (*R*^2^ of 0.7–0.8), so that the TBOAE level at each frequency grows with CEOAE level. The correlations between CEOAEs and TBOAEs were high (often above 0.8) and significant (*p*<0.05). The fits for CEOAEs and TBOAEs for ears with and without SSOAEs were quite similar.

### C. Thresholds of OAEs

**Fig 7:**
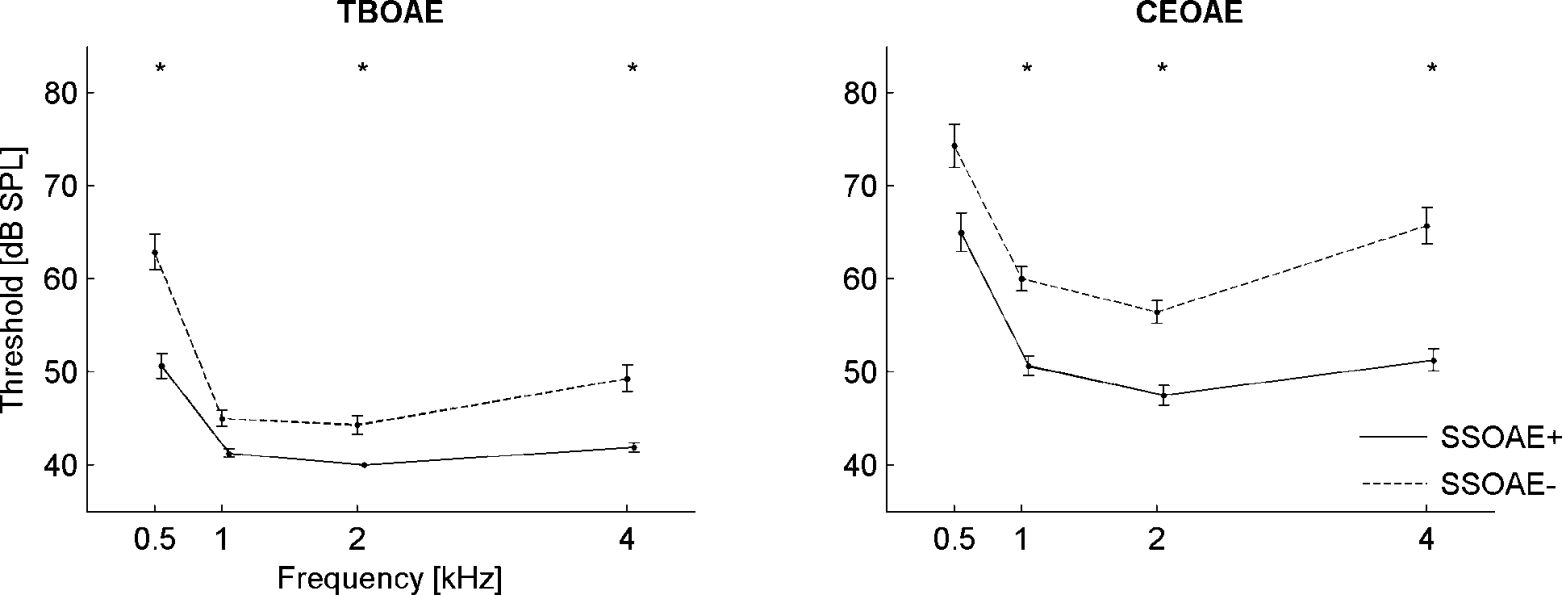
Comparison of mean TBOAE thresholds (left) and CEOAE thresholds (right) for cases with SSOAEs (solid line) and without SSOAEs (dashed line). Measurements were made in half-octave bands for both, and for TBOAEs the stimulus frequency matched the centre frequency of the analysis band. Error bars indicate standard errors; asterisks mark statistically significant differences (*–*p*<0.05).

From the previous I/O plots it is evident that there are distinct differences in the levels at which OAEs from ears with and without measurable SSOAEs cross the noise floor. To analyze these differences in greater detail, the thresholds for each frequency band were calculated and plotted in Fig 7. The OAE threshold as used here is the stimulus level at which the SNR was equal to or greater than 3 dB. The thresholds were calculated for half-octave bands (centered at 0.5, 1, 2, 4 kHz) both for CEOAEs and all TBOAEs. It is apparent from Fig 7 that the thresholds for TBOAEs were much lower than for CEOAEs. Moreover, the thresholds for SSOAE+ ears are lower, both for TBOAEs and CEOAEs. The differences were significant (*p*<0.05) for frequencies of 0.5, 2, and 4 kHz for TBOAEs and 1, 2, and 4 kHz for CEOAEs. They reached 12 dB at 0.5 kHz for TBOAEs, and 14 dB at 4 kHz for CEOAEs. In most cases the thresholds were highest at 0.5 kHz.

### D. Latency of TBOAEs

The latency–intensity functions of TBOAEs for ears with and without SSOAEs for WS and for SL and LL components were investigated. Latencies were calculated only when the response level in a given frequency band exceeded the noise floor by 3 dB.

**Fig 8:**
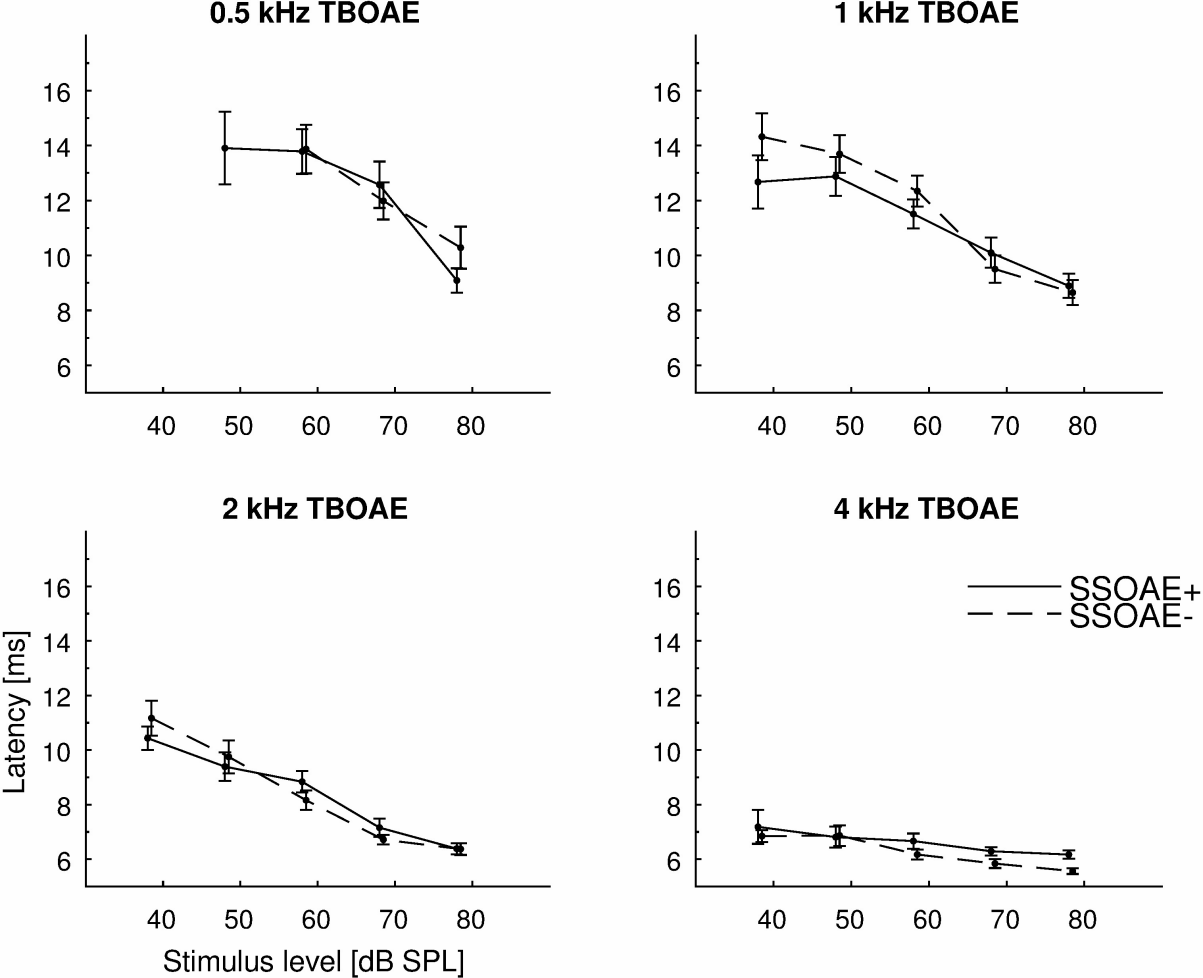
Mean latencies of TBOAEs measured in half-octave bands as a function of stimulus level. The latencies were taken to be the location of the maximum of the whole signal (WS, see Fig 2). Results for 4 different stimulus frequencies are plotted for ears with SSOAEs (solid lines) and without SSOAEs (dashed lines). Error bars indicate standard errors. There were no statistically significant differences between ears with SSOAEs and without SSOAEs for any frequency and stimulus level.

The average functions for WS components are shown at Fig 8. WS components were longest at low stimulus levels, and were shorter for high frequencies and longer for low frequencies. Generally, latencies of WS components were similar for ears with and without SSOAEs.

Separating the SL and LL components is not an easy task. The problem is that different subjects can have a different number of components. Sometimes it could be just one SL and one LL component, which are easy to distinguish. In other cases there can be more components of each type, and choosing which are SL and which are LL becomes arbitrary. This is especially troublesome when analyzing averaged data, as it is problematic to average data which has different numbers of components. Moreover, applying cochlear models to the data is not straightforward. For example, in [9] (their Fig 7) they show that two different models [51,52] do not predict the full range of the latency–frequency relation. Therefore, here the SL and LL components were separated simply by taking the mean value (in ms) of all latencies at the 60 dB level (the mid-point of the latency–intensity curve). The SL components were thus taken to be the components having the highest amplitude over the range 0 ms up to this value. Similarly, LL components were taken to be those whose maximum amplitude appeared between this value and the remainder of the signal. This differentiation was made based on the average envelope for all stimuli levels. The main SL and LL maxima were found and then tracked for envelopes at different stimulus levels. The values of latency used as a criterion to distinguish SL and LL components for TBOAEs at different frequencies were: 13.8 ms for 0.5 kHz, 11.9 ms for 1 kHz, 8.9 ms for 2 kHz, and 6.4 ms for 4 kHz.

**Fig 9:**
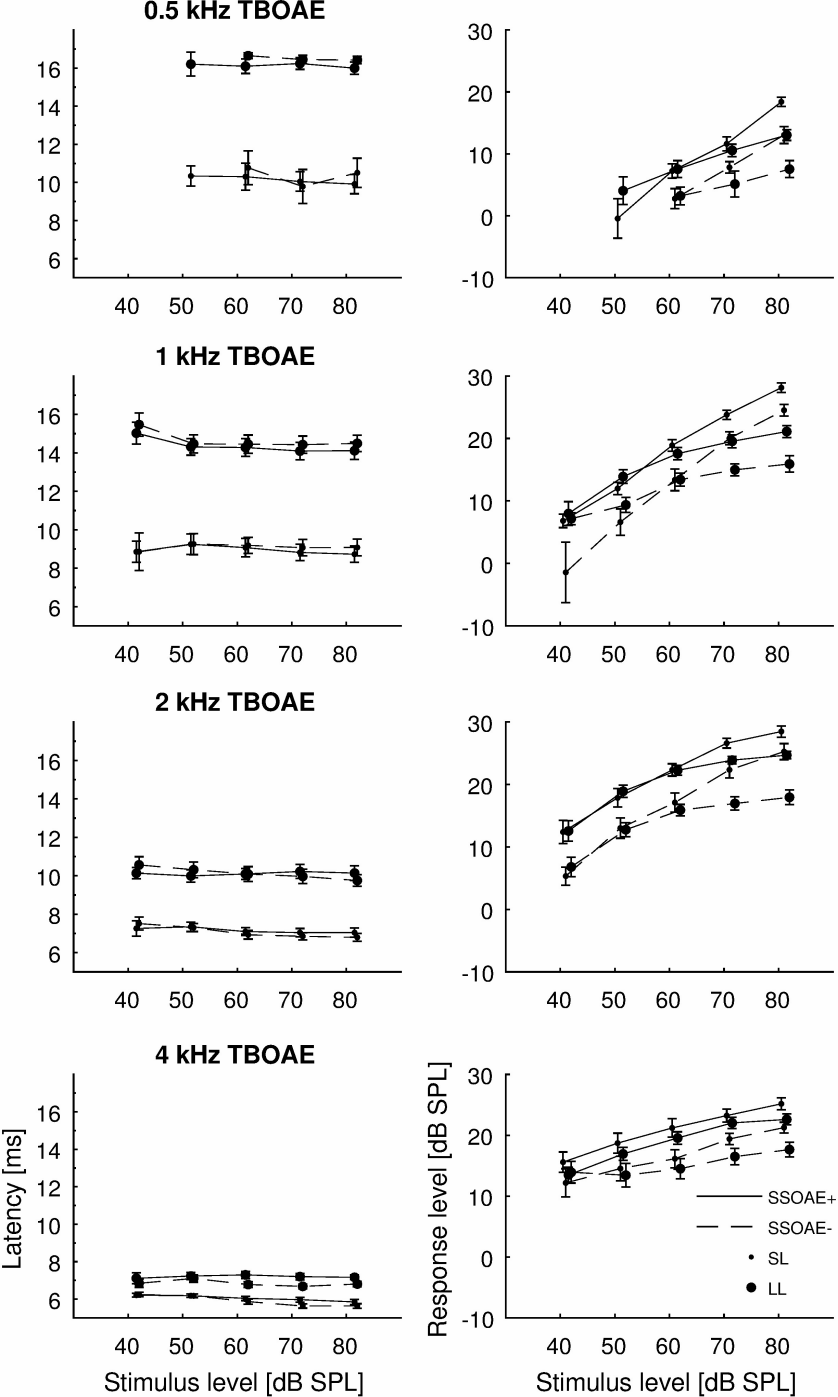
Mean latencies of SL and LL components of TBOAEs (left) and corresponding I/O functions (right). Each row corresponds to a different TBOAE frequency.

Fig 9 shows latencies for SL and LL components and their levels. Unlike WS, both SL and LL were quite stable in time as level increased. WS starts with values close to LL for low stimulus levels and ends with values close to SL for high stimulus levels. Generally, the latencies of all component types were about the same for ears with and without detected SSOAEs, and statistically there were no significant differences between them. Additionally, the I/O functions of the two components were calculated (left column of Fig 9). In this case the amplitude at a given latency was recalculated into dB SPLs. It can be seen that saturation of the LL components occurred at much higher levels than the SL components. Both SL and LL component levels were higher for SSOAE+ ears. Additionally, the difference was higher for LL components, which was not a surprise as it is expected that SSOAE+ ears would have more high amplitude LL components. As LL components are often reflect SSOAEs (e.g. [24]).

## Discussion

The results indicate that TBOAE levels generally depend on whether or not SSOAEs can be detected. Ears in which SSOAEs were detected had higher level TBOAEs than ears where they were absent, and this effect was evident at most of the stimulus levels used in the study. The inference is that ears with SSOAEs above the noise floor have lower TBOAE detection thresholds.

The I/O functions of TBOAEs were found to be quite similar to those of CEOAEs, but showing less tendency to saturate. This result is similar to DPOAEs, which also tend to saturate less easily than CEOAEs [28]. Lewis and Goodman [43] also found a similar lack of saturation of TBOAEs. Related studies have also shown that some individual subjects exhibit saturation and others do not [53,54]. However, generally it has been reported that TBOAEs have, on average, very similar I/O characteristics to CEOAEs [30,41].

To date, I/O characteristics of TBOAEs for ears with and without SOAEs have only been reported for 1.5 kHz TBOAEs [30]. In that study, a slightly different stimulus intensity range was used, but nevertheless the results were similar to the present study, and showed that SOAE+ ears exhibit higher levels and lower thresholds. There are also some findings for CEOAEs [34] and DPOAEs [28,35] in which slightly different analysis methods were used for each OAE. In the first, only global CEOAE values were used; in the second, DPOAEs close to SOAE frequencies were studied. In both cases quite a large difference emerged between ears with and without SOAEs across all stimulation levels. A similar picture emerged here for TBOAEs: differences were pronounced when measured globally, but when analyzed into frequency bands they were relatively small. The conclusion is that the presence of an SSOAE has a larger effect on the global signal than in a particular frequency band because in each case the SSOAEs originate from different cochlear locations.

The presence of SSOAEs also affects TBOAE thresholds, although of course the definition of threshold will change the results. Here, the threshold was defined as an SNR ≥3 dB, which is a slightly more strict criterion than that used by [21] of reproducibility ≥50%. Reproducibility and SNR are directly related, so that an SNR of 3 dB corresponds to a reproducibility of 67%. Previously, thresholds have been documented only for global values of CEOAEs and TBOAEs [21, 30]. In the present study, they were analyzed for half-octave bands around four center frequencies (0.5, 1, 2, 4 kHz). It was found that TBOAEs have lower detection thresholds than CEOAEs, and that ears with SSOAEs had lower thresholds, both for CEOAEs and TBOAEs. Thresholds for both TBOAEs and CEOAEs were lowest at 2 kHz and highest for 0.5 kHz. However, the estimates shown here should be treated with caution: averaging of all stimuli ended after a maximum of 780 averages, and longer averaging produces lower thresholds. Moreover, the lowest stimulus level provided by the system was 40 dB SPL, so thresholds could not be lower than that.

The frequency band around 0.5 kHz was particularly interesting. Results presented here indicate that 0.5 kHz TBOAEs are low level in SSOAE–ears. In some ears, only 80 dB pe SPL tone bursts produced a response that exceeded the half-octave band noise level. Once again, this limitation could be overcome by using a higher number of averages (e.g. [55]). At 0.5 kHz, both TBOAEs and CEOAEs have high detection thresholds; i.e. compared with responses at higher frequencies, the responses require high stimulus levels in order to exceed the noise. This is mainly due to especially low response levels at 0.5 kHz. They were lower on average by 10 dB compared to response levels for 1, 2, and 4 kHz tone bursts. This finding might be related to the low-frequency filter settings of the recording system [56,57]. It might also depend on the width of the data window. At these low frequencies, the long latencies might cause the responses to fall outside the 20 ms window used here. As a result, it is possible that the LL components of TBOAEs beyond 20 ms might have been recorded as SL components in the subsequent buffer. This is a potential artifact associated with the use of a 20 ms window. However, it was not possible to investigate this further as the ILO equipment does not allow the recording window to be lengthened.

In the current paper there were no differences in TBOAE latency between ears with and without SOAEs. However, the latency was shorter for the higher frequency stimuli. The WS latency was also shorter for higher stimulus levels. This effect was not related to gradual shifts in latency but rather a switch to different maxima in the signal envelope, as shown in Fig 2. This was also shown for the whole group when components were grouped into SL and LL. SL components are usually related to the first maximum of the TBOAE waveform at the highest stimulation level (here 80 dB pe SPL), while LL are usually related to the maximum at lower stimulation levels. Latencies of both SL and LL were quite stable for different stimulation levels for all tone burst stimuli irrespective of frequency. The same effect was found earlier for 2 kHz TBOAEs [43] and also for CEOAEs [42]. The latencies of SL and LL were similar in ears with and without SSOAEs. However, similarly to global and half-octave levels, there were differences in levels of SL and LL components between ears with and without SSOAEs. One might expect that LL components would show higher levels for ears with SSOAEs, but interestingly the finding was that SL components also had higher levels in ears with SSOAEs. So the presence of spontaneous components also promotes the generation of SL components, which are likely to be ‘purely’ evoked components. The results here confirm previous findings [43] that LL components are mostly responsible for saturation of response with increasing level. In summary, even though the presence of SSOAEs had a clear effect on the response levels of the SL and LL components, no effect on their latencies was evident.

The latency of TBOAEs depends on the length of the tone burst stimulus [9,43]; however, here fixed stimulus lengths were used. The fixed length means that our results should be easy to reproduce, providing a reference point for researchers and clinicians using the same system. In some previous work the tone-burst stimuli were exceptionally long (up to 8–10 ms for 0.5 kHz), resulting in correspondingly long estimates of TBOAE latency [7,40,51]. In such cases, it is difficult to compare the findings with those from the present work, which used comparatively short bursts (2 ms). Nevertheless, the results from the present research are in line with earlier work [9,43] where latency as a function of stimulus level was also calculated (although only results for 2 kHz TBOAEs were shown). In the first paper [9], 2 ms tone burst were also used (among others, see their Fig 9), the same as the present work. Their findings were a latency of about 8 ms for 40 dB stimulation and about 4 ms for 80 dB. In comparison, the findings here are around 10 ms for 40 dB and 6 ms for 80 dB. However, here a relationship between stimulus level and latency formed nearly straight line, similar to another study [43]; note, however, that the earlier work used slightly different bursts (3 cycles), and the 2 kHz TBOAE latencies were slightly shorter than here.

It appears that TBOAEs are affected by the presence of SSOAEs, even when the underlying spontaneous activity is not close in frequency to the evoked components. This is especially pronounced for the 0.5 kHz TBOAEs which are well below the frequency of most SSOAEs. In addition, both the SL and LL components appear affected by the presence of SSOAEs. This points to an explanation in which a cochlea that emits SOAEs is likely to be more generally active, including having an enhanced level of TBOAEs. For example one well-accepted model depicts the cochlea as an acoustic resonator [58], in which case the presence of spontaneous activity would imply higher cochlear reflectance. Such a cochlea would be more active over its entire range, not just at the frequency of a spontaneous emission. One consequence would be that evoked OAEs, such as CEOAEs and TBOAEs, would have higher amplitudes in ears with SOAEs than in ears without SOAEs.

Finally, some limitations of the present study should be mentioned. Most of limitations come from using the ILO system, and its inability to tailor stimuli and adjust OAE acquisition protocols. Therefore the settings described in the materials and method section (e.g. very short bursts, short acquisition window, non-linear recording mode only) were set by the device and not chosen by the authors. Although this is a major limitation, it does have the considerable advantage of easy reproducibility: the ILO 292 is quite a popular system and the results set out here may provide a useful benchmark for other users. And though a large number of averages was employed here, it would undoubtedly be better to use an even greater number. This consideration is particularly important when low-level stimuli are used, and although it would be of interest to investigate responses to very low-level stimuli, 40 dB is the lowest available from the ILO system. One additional issue to be kept in mind is that SOAEs might interact with stimuli in various ways, for example be synchronized (as in SSOAEs) or be suppressed by the stimulus itself [59]. The latter situation is the inverse to what was investigated here, and would require a somewhat different approach to understand properly.

## Conclusions

TBOAEs are significantly affected by the presence of SSOAEs, even though TBOAEs reflect activity from a narrower region of the cochlea than do CEOAEs. In ears with SSOAEs, TBOAEs show higher levels and lower detection thresholds. TBOAEs might be a better choice than CEOAEs when testing subjects with low-amplitude OAEs or in ears without SSOAEs. Interestingly, the presence of SSOAEs seems not to affect TBOAE latency. This proves that latency is quite a robust parameter and supports its use when studying evoked OAEs. TBOAEs share many of the features of CEOAEs, but they have the useful property of providing additional information in the lower frequency range, e.g. at 0.5 kHz where CEOAEs show responses only at 80 dB SPL.

## Supporting information

S1 File Individual data for each Fig. (XLSX)
